# Rapid Escalation of High-Volume Exercise during Caloric Restriction; Change in Visceral Adipose Tissue and Adipocytokines in Obese Sedentary Breast Cancer Survivors

**DOI:** 10.3390/cancers13194871

**Published:** 2021-09-28

**Authors:** Carol J. Fabian, Jennifer R. Klemp, Nicholas J. Marchello, Eric D. Vidoni, Debra K. Sullivan, Jennifer L. Nydegger, Teresa A. Phillips, Amy L. Kreutzjans, Bill Hendry, Christie A. Befort, Lauren Nye, Kandy R. Powers, Stephen D. Hursting, Erin D. Giles, Jill M. Hamilton-Reeves, Bing Li, Bruce F. Kimler

**Affiliations:** 1 Department of Internal Medicine, Division of Medical Oncology, University of Kansas Medical Center, 3901 Rainbow Boulevard, Kansas City, KS 66160, USA; cfabian@kumc.edu (C.J.F.); jklemp@kumc.edu (J.R.K.); jlnydegger@gmail.com (J.L.N.); tphillip@kumc.edu (T.A.P.); akreutzjans@kumc.edu (A.L.K.); lnye@kumc.edu (L.N.); kpowers@kumc.edu (K.R.P.); 2 Department of Nutrition, Kinesiology, and Psychological Sciences, University of Central Missouri, P.O. Box 800, Warrensburg, MO 64093, USA; marchello@ucmo.edu; 3 Department of Neurology, University of Kansas Medical Center, 3901 Rainbow Boulevard, Kansas City, KS 66160, USA; evidoni@kumc.edu (E.D.V.); bhendry@kumc.edu (B.H.); 4 Department of Dietetics and Nutrition, University of Kansas Medical Center, 3901 Rainbow Boulevard, Kansas City, KS 66160, USA; dsulliva@kumc.edu (D.K.S.); jhamilton-reeves@kumc.edu (J.M.H.-R.); 5 Department of Population Health, University of Kansas Medical Center, 3901 Rainbow Boulevard, Kansas City, KS 66160, USA; cbefort@kumc.edu; 6 Department of Nutrition, Nutrition Research Institute, University of North Carolina at Chapel Hill, 235 Dauer Drive, Chapel Hill, NC 27599, USA; hursting@email.unc.edu; 7 Department of Nutrition, Texas A&M University, 214 Cater-Mattil 2253 TAMU, 373 Olsen Blvd, College Station, TX 77843, USA; egiles@tamu.edu; 8 Department of Urology, University of Kansas Medical Center, 3901 Rainbow Boulevard, Kansas City, KS 66160, USA; 9 Department of Pathology, University of Iowa, 200 Hawkins Dr, Iowa City, IA 52242, USA; bing-li@uiowa.edu; 10 Department of Radiation Oncology, University of Kansas Medical Center, 3901 Rainbow Boulevard, Kansas City, KS 66160, USA

**Keywords:** biomarkers, breast cancer prevention, DXA, exercise, visceral adipose tissue

## Abstract

**Simple Summary:**

Aerobic exercise reduces risk for developing breast cancer or for breast cancer recurrence. In obese women exercise can significantly augment the effects of caloric restriction on visceral fat, reducing metabolic abnormalities and cancer. Women who are older, obese, and sedentary, especially those who have been treated for breast cancer, find it difficult to initiate and achieve the minimum or optimum levels of exercise. In a two-part pilot we found that by providing older, obese, sedentary breast cancer survivors 12 weeks of twice weekly personal training sessions, they could safely increase exercise to ≥200 min/week by 9 weeks during caloric restriction. At 24 weeks, high levels of exercise were still observed with continued behavioral support and study-provided exercise facility. Substantial improvement in visceral fat and breast cancer risk biomarkers were observed with this affordable intervention that is readily exportable to the community.

**Abstract:**

Aerobic exercise reduces risk for breast cancer and recurrence and promotes visceral adipose tissue (VAT) loss in obesity. However, few breast cancer survivors achieve recommended levels of moderate to vigorous physical activity (MVPA) without supervision. In a two-cohort study, feasibility of 12 weeks of partially supervised exercise was started concomitantly with caloric restriction and effects on body composition and systemic risk biomarkers were explored. In total, 22 obese postmenopausal sedentary women (including 18 breast cancer survivors) with median age of 60 and BMI of 37 kg/m^2^ were enrolled. Using personal trainers twice weekly at area YMCAs, MVPA was escalated to ≥200 min/week over 9 weeks. For cohort 2, maintenance of effect was assessed when study provided trainer services were stopped but monitoring, group counseling sessions, and access to the exercise facility were continued. Median post-escalation MVPA was 219 min/week with median 12-week mass and VAT loss of 8 and 19%. MVPA was associated with VAT loss which was associated with improved adiponectin:leptin ratio. In total, 9/11 of cohort-2 women continued the behavioral intervention for another 12 weeks without trainers. High MVPA continued with median 24-week mass and VAT loss of 12 and 29%. This intervention should be further studied in obese sedentary women.

## 1. Introduction

In observational studies, self-reported moderate to vigorous physical activity (MVPA), i.e., aerobic exercise, has been associated with reduced risk for breast cancer [1,2,3,4,5], and overall mortality after a cancer diagnosis [6,7]. A recently reported prospective study performed in conjunction with a large adjuvant trial indicated meeting MVPA guidelines before and after a breast cancer diagnosis was associated with reduction in breast cancer recurrence and cancer mortality [8]. Benefits for aerobic exercise on breast cancer outcomes likely accrue via improvements in adipokine profile, insulin sensitivity, mitochondrial and immune function as well as reduction in chronic inflammation [9].

2.5–5 h/week of recreational physical activity is recommended for general health, with a minimum of ≥150 min of at least moderate intensity aerobic activity/week for breast cancer survivors [10,11]. Unfortunately, only a minority of women at increased risk for breast cancer or breast cancer survivors report meeting even minimum aerobic guidelines [11,12,13]. Obese survivors are least likely to meet minimum activity guidelines [13], despite being more likely to exhibit a metabolic risk profile which could be improved with exercise [14,15,16,17,18]. Higher minimum aerobic exercise volumes (MVPA ≥ 200 min/week) are recommended for women attempting weight loss and maintenance [19,20]. Individuals achieving and maintaining a 30-pound loss report an hour of exercise daily [21]. Although randomized trials have had conflicting results, those in which actual exercise was measured with an accelerometer have generally shown favorable long-term results with higher exercise volumes [22,23,24,25]. 

Barriers to exercise include lack of resources and skills, as well as fear of injury in older individuals; thus, sedentary postmenopausal obese women may benefit from initial in-person training [26]. Trials achieving accelerometer-documented high volume MVPA in breast cancer survivors have used partial or fully supervised exercise for the duration of the study [27,28]. Long-term supervised exercise with personal trainers may not be feasible for many women outside of a clinical trial, but short-term supervision for exercise initiation and ramp-up might be exportable to the community as part of a rehabilitation plan for breast cancer survivors or prevention of breast cancer in those at increased risk for development of the disease. 

**Figure 1:**
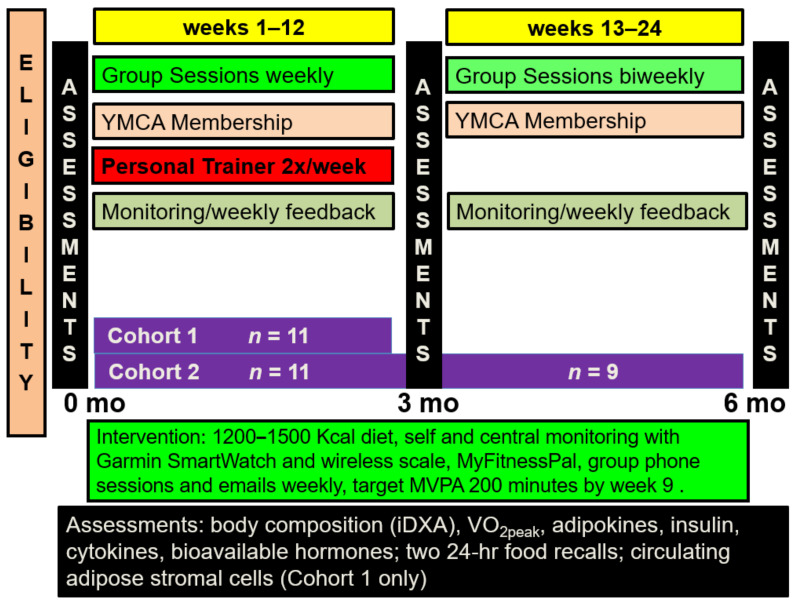
Diet and exercise interventional design for the two cohorts.

Our goal was to first assess feasibility and risk biomarker effects of rapid escalation of partially supervised MVPA to ≥ 200 min/week during the initial 9 weeks of a 12-week behavioral weight loss intervention in obese sedentary postmenopausal breast cancer survivors. The intervention consisted of twice weekly personal trainer sessions for 12 weeks, a reduced calorie diet, self- and central monitoring via a Garmin Vivoactive™ smartwatch, Garmin Connect™, a wireless scale, and MyFitnessPal™, and a weekly group phone call with 10–12 women who start the program at the same time (Figure 1). If MVPA goals appeared feasible in the initial cohort, we sought to confirm the initial 12-week results and then further assess if MVPA could be maintained without a personal trainer but with continued exercise facility membership and behavioral support. Since a key portion of the behavioral component involved a cohort of subjects initiating this intervention together, the second group included four high risk women as well as seven cancer survivors to facilitate timely intervention start. 

Risk biomarkers assessed included systemic levels of adipocytokines, insulin, bioavailable hormones, anthropomorphic measures and body composition and fitness as assessed by VO_2peak_; of particular interest was visceral adipose tissue (VAT). Excess VAT and liver adipose tissue are thought to be disproportionately responsible for the metabolic abnormalities that link obesity with postmenopausal breast cancer risk and recurrence as well as cardiovascular disease [29,30,31]. MVPA enhances VAT loss achieved with dietary caloric restriction [32,33]; but even without caloric restriction, aerobic physical activity reduces VAT [34] via enhanced lipolysis and fatty acid oxidation [32,35,36,37,38]. A ≥25% reduction in VAT has been shown to improve insulin sensitivity, adipokine profile, and inflammatory markers [39]. A ≥25% VAT reduction is often observed in those with a ≥10% weight loss which in turn has been reported to improve systemic risk biomarkers for breast cancer [40] and, if sustained, a reduction in risk for breast cancer [41] as well as cardiovascular events [42]. VAT can now be conveniently measured via dual-x-ray absorptiometry, providing similar results as computed tomography (CT) and MRI without the radiation (CT) and at a fraction of the cost [43]. VAT measured by DXA correlates with metabolic measures [44] and a DXA-measured VAT of ≥1.2 kg is associated with increased risks of metabolic syndrome [43]. VAT is replacing BMI in obesity-related cardiovascular research, although to date it has received minimal attention in cancer research [44,45,46]. 

## 2. Materials and Methods

### 2.1. Eligibility

Women were consented to cohort 1 (NCT02963740; registered 16 November 2016) or cohort 2 (NCT03720111; registered 30 August 2017) prior to initiation of baseline testing through the University of Kansas Medical Center (KUMC) Breast Cancer Prevention and Survivorship Research Center. Women were required to have had either a prior diagnosis of breast cancer (cohorts 1 and 2) or increased risk for development of breast cancer (cohort 2). All women within a cohort were started on the intervention at the same time. Cohort 2 allowed high risk women (≥2-fold increase in Tyrer-Cuzick 10-year risk for age group) such that women in that cohort could be started on the intervention in a timely fashion after identification. Eligibility for both studies included postmenopausal status, BMI between 30 and 45 kg/m^2^, <60 min of self-reported exercise per week but able to walk ≥30 min on a level surface, and completion of any chemotherapy or radiation at least 3 months prior to intervention start. Women were ineligible if they had undergone prior bariatric surgery, were on metformin or weight-loss medications, or had participated in a structured weight loss intervention within 6 months. 

### 2.2. Baseline Studies

#### 2.2.1. Anthropomorphic Measures and Body Composition

Weight, height, waist circumference (below lowest rib) [47], and body composition by dual x-ray absorptiometry (GE Lunar iDXA, GE Healthcare, Chicago, IL, USA) were measured in a hospital gown at baseline and after 12 weeks of intervention. iDXA coefficient of variation (CV) is <1% for total mass and fat, and 5.4% for VAT for overweight and obese individuals [48]. 

#### 2.2.2. Cardiopulmonary Fitness Testing-Peak Oxygen Consumption (VO_2peak_)

Cardiovascular fitness was measured as peak oxygen consumption (VO_2peak_) during a graded exercise test with a modified Bruce Ramp Treadmill protocol with a Parvomedics Truetone™ (ParvoMedics, Salt Lake City, UT, USA) 2400 system at baseline and after 12 weeks of intervention. Participants were encouraged to walk until they reached volitional exhaustion. For women ages 50–59 a VO_2peak_ of <20.2 and 20.2–22.7 mL/kg/min was considered very poor and poor, respectively. Corresponding values for women ≥60 were <17.5 and 17.5–20.1 mL/kg/min [49]. We assessed change in fatigue by the Brief Fatigue Inventory [50], reasoning that fatigue is the most likely of the quality-of-life indices to be affected by improved physical activity.

### 2.3. Behavioral Weight Loss Intervention

In two in-person group sessions, initial instruction was provided in the use of personal monitoring tools (Garmin Vivoactive™ wrist type activity tracker that also measures heart rate, MyFitnessPal™, and wireless digital scale) and reduced calorie diets. Women received a membership at area YMCAs, twice weekly individual sessions with a research-certified personal trainer, weekly individual feedback summary based on electronic data in Garmin Connect™ (C.J.F.) and a weekly group phone behavioral meeting. Weekly group phone calls were led by a registered dietitian (N.J.M.) or clinical health psychologist (J.R.K.) with input from two oncologists (C.J.F., L.N.). Calls centered on diet and exercise behavior education, goal setting, and discussion of barriers and solutions. A manual focused on nutrition, physical activity, and issues of special interest to cancer survivors or women at increased risk for the disease, was also provided (J.R.K. and C.A.B.). 

### 2.4. Diet

Daily meal recommendations were two pre-packaged entrees ≤350 calories/entree, 2–3 low calorie high protein snacks, and 5 servings of fruits and vegetables per day (all purchased by participants). Diets were designed to provide 1200–1500 kcal (~ 20% reduction from requirements) with <30% kcal from fat and a minimum of 60 g protein per day, in line with current recommendations [51]. Caloric intake could be adjusted on an individual basis depending upon weight loss. MyFitnessPal™ was used by participants to self-monitor caloric intake and diet composition. Caloric intake was assessed via two 24 h dietary recalls (one weekday and one weekend day) performed at baseline and at 12 weeks (both cohorts) and at 24 weeks (cohort 2). The food recalls were then analyzed using the Nutrition Data System for Research (NDSR, University of Minnesota) software.

### 2.5. Physical Activity Escalation and Measurements 

Planned total physical activity was started at 100 min/week for the first 4 weeks and was to be escalated by 25 min/week to 300 total min/week by week 9 with ≥200 min at ≥moderate intensity. Women had individual sessions twice weekly for 12 weeks with research-certified personal trainers. Trainer session duration was 20 min per session at week one and was escalated by 10 min per session each week to a maximum of 50–60 min per session. Exercise was primarily walking on a treadmill at first, but then different activities were incorporated by individual trainers and participants. Women were also asked to exercise at home (or unsupervised at the YMCA) for similar durations (as per supervised sessions) an additional 2–3 times per week; thus, 40% of weekly aerobic exercise goals were to be supervised and 60% unsupervised during weeks 1–12. Exercise intensity was initiated at 40–60% of heart rate reserve during weeks 1–4, based on previous experience of our exercise physiology team member (E.D.V.) with older poorly fit individuals [52]. Intensity was subsequently escalated to 45–80% heart rate reserve consistent with moderate to vigorous activity recommendations for older women [53]. The study-provided YMCA memberships gave access to all YMCA facilities and classes in the Kansas City area. 

Volume and intensity of physical activity was measured via study-provided Garmin Vivoactive™ smart watch accelerometers which also recorded heart rate. These devices were worn day and night and were removed for charging only. Participants were also supplied with polar monitor type chest straps (worn at minimum during supervised exercise) to verify the accuracy of the wrist devices. To facilitate achievement of appropriate exercise intensity, watches were individually programmed with heart rate reserve levels determined from baseline exercise testing. “Zone 3” as visualized on the watches was translatable to 45–60% heart rate reserve, Zone 4 was 61–80% and Zone 5 was 80–100% of heart rate reserve. Women were encouraged to check their watches during activity to make sure they were exercising in Zone 3 or 4 as appropriate. Garmin Vivoactive Smart Watches with heart rate monitoring capability have been shown to underestimate total steps by ~9% and overestimate MVPA by an absolute percentage of ~6%, with better accuracy at higher speed [54].

Activity and caloric intake from MyFitnessPal™ were recorded centrally via Garmin Connect™, computed by a research coordinator and reported to study participants along with weight prior to the weekly group behavioral meeting. 

As a measure of MVPA achievement for each participant after 12 weeks, the median of weekly MVPA values for weeks 9–12 (post-escalation phase) was computed. 

### 2.6. Systemic Biomarkers

Fasting blood was obtained at baseline and after 12 weeks of intervention. Samples were processed to serum and frozen in aliquots at −80 °C. All samples from the same individual were assayed in duplicate in the same run in the Breast Cancer Prevention Center Laboratory (TP). The exception was for buffy coat specimens obtained from cohort 1 women only, which were processed immediately in the Breast Cancer Prevention Laboratory and assessed same day in the Flow Cytometry Core Laboratory for circulating adipose stromal cells [55]. Luminex Milliplex^®^ Human Adipokine Magnetic Bead Panels 1 & 2 (HADK1 & 2 MAG-61K, Millipore Sigma, Burlington, MA, USA) were used to assay adiponectin, resistin, total PAI-1, lipocalin-2, leptin, insulin, tumor necrosis factor alpha (TNFα), hepatocyte growth factor, interleukin-6, and macrophage chemotactic protein-1. Adiponectin and leptin were also assessed by enzyme-linked immunosorbent assay (ELISA) (R&D Systems #DRP300 and R&D Systems #DLP00, Minneapolis, MN, USA). Fibroblastic growth factor-2 and fibroblastic growth factor-21 were assayed by ELISA (R&D Systems #DFB50, R&D Systems #DF2100). ELISA was used for lysyl oxidase (#MBS 2023535 MyBioSource Inc., San Diego, CA, USA) and fatty acid binding protein 4 (#10007614 Cayman Chemical; Ann Arbor, MI). ELISA was also used for estradiol (CAN-E- 430), testosterone (CAN-TE-250), sex hormone binding globulin (CAN-SHBG-410), and high sensitivity C-reactive protein (CAN-CRP-4360) all from Diagnostics Biochem Canada; Dorchester, ON, Canada. Enzyme immunoassay (EIA) was used to assess omentin-1 and visfatin (#EIA-OME and #EIA-VIS RayBiotech; Norcross, GA, USA) 

### 2.7. Statistical Analysis

Non-parametric methods (SPSS, version 24, IBM, Armonk, NY, USA) were used for all analyses due to the small sample sizes and the fact that not all variables were normally distributed. The Wilcoxon signed-rank test for paired samples was used for assessment of change in variables over the course of the intervention. The Mann–Whitney test was used for comparison between groups. Associations between changes in the biomarkers and weight change were evaluated by Spearman test and linear regression analysis. *p* < 0.05 was considered statistically significant with no adjustment for multiple comparisons. Datasets analyzed are available on reasonable request. 

## 3. Results

### 3.1. Baseline Characteristics

Twenty-three women underwent baseline testing for the diet and exercise intervention (12 for cohort 1 and 11 for cohort 2). One woman screened in cohort 1 did not have a satisfactory baseline VO_2peak_, resulting in 11 participants for each cohort. The 18 breast cancer survivors and 4 high risk women had similar baseline anthropomorphic, body composition, and cardiopulmonary testing results (Table 1); therefore, baseline characteristics are combined for descriptive purposes. 

All 22 women self-reported <60 min exercise per week at baseline. All women were considered obese by BMI (≥30 kg/m^2^ eligibility requirement) and by fat mass index (≥13 kg/m^2^); and centrally obese by waist circumference (≥88 cm) [47]. Median BMI was 37 kg/m^2^, and median abdominal girth was 107 cm. Median fat mass index was 17.6 kg/m^2^ and median VAT by iDXA was 1.7 kg; all but one woman had a VAT ≥1.2 kg, placing them at increased risk for metabolic syndrome [39,45]. Baseline BMI, total mass, total fat, and fat mass index were all highly correlated (*p* < 0.001) with each other, but baseline VAT was not significantly correlated with any of these. Baseline median VO_2peak_ was 18.7 mL/kg/min, and 18/22 women (14 survivors and all 4 high-risk women) had a fitness level rated as very poor to poor for age group. For the 18 breast cancer survivors, a median of 27.5 months had elapsed since diagnosis. In total, 55% had received prior chemotherapy, and 55% were on current endocrine therapy (predominately aromatase inhibitors).

### 3.2. Adherence and Adverse Events 

The Garmin Vivoactive™ activity trackers with heart rate monitoring capability were worn continuously except for charging. In total, 19/22 women wore their activity trackers for ≥95% of the 84 days of the initial 12 weeks. Central monitoring was accomplished via Garmin Connect™ for weight, steps, volume, and intensity of physical activity; as well as caloric intake derived from MyFitnessPal™. Attendance at trainer sessions was recorded by both participant and trainer. Attendance at group phone meetings was taken by roll call and recorded by the leader at the beginning of each meeting. For the 22 women in cohorts 1 and 2 combined, there was excellent adherence over the initial 12 weeks with no difference between survivors and high-risk women. There was a median of 83% attendance for the 24 possible trainer sessions (range of 4–24), median 83% attendance at 12 possible weekly group phone calls (range 4–12) and 81% compliance with food intake recording (defined as reporting at least 900 kcal consumed daily). There were no serious adverse events related to exercise and no individuals dropped out in the initial 12 weeks. Seven individuals reported grade 1 or 2 events possibly or probably related to exercise such as back or elbow pain. The 11 women in cohort 2 were offered a second 12 weeks of intervention during which the study-provided trainer was discontinued but behavioral support and YMCA memberships were continued. Two breast cancer survivors dropped out after the initial 12 weeks but prior to 24 weeks due to personal commitments.

### 3.3. Change in MVPA and Cardiorespiratory Fitness and Quality of Life Assessment 

MVPA recorded in Garmin Connect™ increased from a median of 55 min during week 1 to a median of 219 min/week during weeks 9–12 (the post-escalation phase). In total, 17/22 (77%) achieved an MVPA of ≥200 min/week during any one week during the initial 12-week period and 13/22 participants achieved a median of ≥200 min/week during weeks 9–12. Most (87%) of the MVPA was of moderate intensity (45–60% heart rate reserve; Table 2). After 12 weeks of the intervention VO_2peak_ increased from a median baseline of 18.9 mL/kg/min to 22 mL/kg/min (median relative increase of 13%; Table 2). There was no difference in achieved MVPA or VO_2peak_ between group as a whole or if restricted to the 18 survivors (Table 3). As expected, the Brief Fatigue Inventory demonstrated an improvement in the majority of women. The mean score for fatigue interfering with six common activities improved from a median of 1.5 at baseline to a median of 0.4 at 12 weeks, with a median change over the interval of −0.6 (*p* = 0.018, Wilcoxon test).

Nine of the 11 women in cohort 2 completed an additional 12 weeks of the behavioral intervention which included continued study-provided access to the exercise facility but omission of the trainer after week 12. The 24-week median MVPA was 189 min/week (Table 4). Median baseline VO_2peak_ for these women was 18 mL/kg/min, 21.7 mL/kg/min at 12 weeks, and 22.9 mL/kg/min at 24 weeks (26% relative increase from baseline). 

### 3.4. Change in Diet 

Caloric intake was estimated from two 24 h dietary recalls performed at baseline and after 12 weeks for cohorts 1 and 2, and after 24 weeks for cohort 2. Women in cohort 1 were asked to complete recalls on their own using the National Cancer Institute (NCI) Automated Self-Administered (ASA24^®^) Dietary Assessment Tool; however, due to difficulties in utilizing the online data entry system, the results were not considered to be reliable and were therefore not used for analysis. As an adjustment, women in cohort 2 completed the recall with assistance provided by trained dietitians from the Nutrition Shared Resource staff of the University of Kansas Cancer Center. For cohort 2, median change in daily caloric intake from baseline was estimated at −350 kcal for the initial 12 weeks and was maintained through 24 weeks. 

### 3.5. Change in Weight and Body Composition

**Figure 2:**
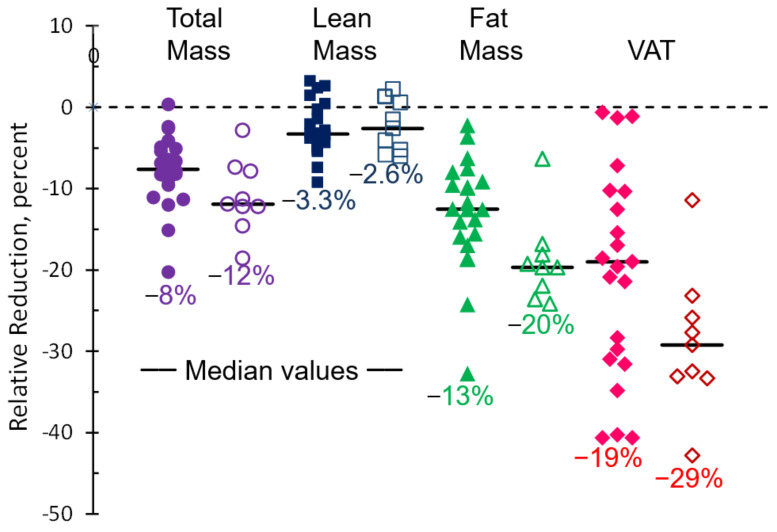
Change in body composition after 12 weeks (closed symbols) for 22 participants in cohorts 1 and 2; and 24 weeks (open symbols) for 9 participants in cohort 2.

At 12 weeks, considering the 22 women in both cohorts together, median relative change was −8% for total mass; −13% for total fat; −19% for VAT; and −3% for lean mass (Figure 2 and Table 2). Total fat was linearly correlated with mass loss (R^2^ ≥ 0.72) but VAT loss was not (R^2^ = 0.29). The proportion of individuals with an increased risk of metabolic syndrome based on iDXA VAT ≥ 1.2 kg decreased from 21/22 (95%) to 13/22 (59%). 

Similar results after 12 weeks were observed when restricting analysis to the 18 breast cancer survivors (Table 3). 

For the nine women in cohort 2 completing 24 weeks, body composition change from baseline were −12% for total mass, −20% for total fat, −29% for VAT, and −3% for lean mass (Figure 2 and Table 4). 

### 3.6. Association of Change in Fitness and Body Composition with Achieved MVPA 

**Figure 3:**
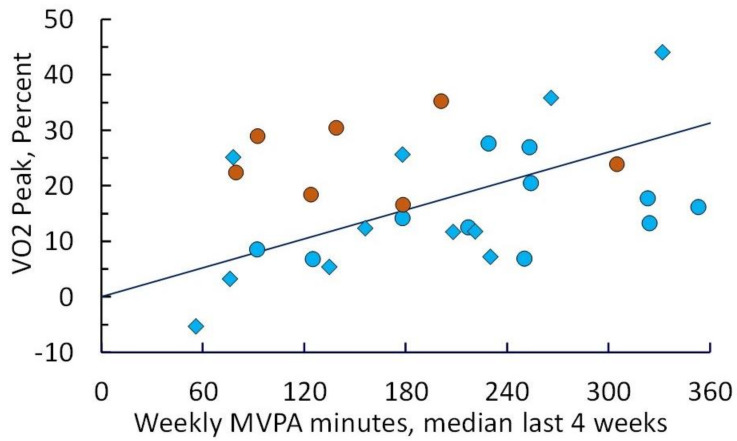
Improvement in cardiopulmonary fitness after 12 and 24 weeks of intervention as measured by peak oxygen consumption (VO_2peak_) relative to baseline, plotted as a function of weekly MVPA achieved (median of recorded values weeks 9–12 or weeks 21–24). Diamonds denote participants in cohort 1; circles denote participants in cohort 2 (blue = 12-week values; orange = 24-week values). Linear regression yields a slope of 0.057% change in VO_2peak_ per min of MVPA (*p* = 0.021).

Median post-escalation phase MVPA during weeks 9–12 for both cohorts and for cohort 2 only during weeks 21–24 was correlated (Spearman’s rho) with improvement in VO_2peak_ (*p* = 0.004) (Figure 3). 

**Figure 4:**
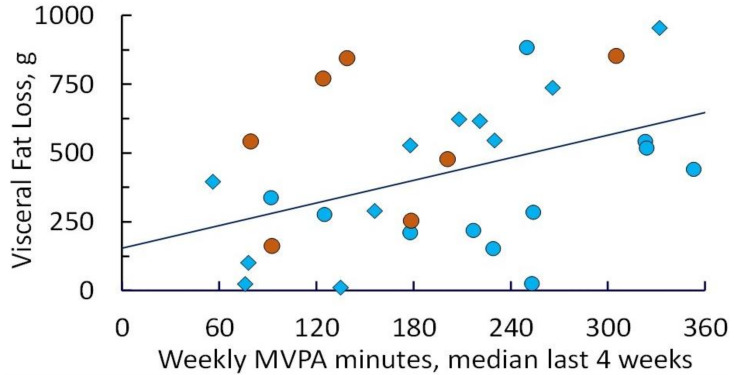
Visceral fat lost (percent relative to baseline) as assessed by iDXA after 12 and 24 weeks of intervention, plotted as a function of weekly MVPA achieved (median of recorded values for weeks 9–12 or weeks 21–24). Diamonds denote participants in cohort 1; circles denote participants in cohort 2 (blue = 12-week values; orange = 24-week values). Linear regression yields a slope of 0.063% visceral fat loss per min of MVPA (*p* = 0.011).

MVPA was also associated with reduction in total mass and fat (*p* ≤ 0.001), and VAT loss (*p* = 0.015) at both 12 and 24 weeks. There was a linear correlation of VAT loss with MVPA (Figure 4), yielding a slope of 0.063% visceral fat loss per min of MVPA that was statistically significantly different than zero (*p* = 0.011). 

Eight of the 22 women achieved a ≥25% VAT loss after 12 weeks of intervention; these 8 women had a median of 258 MVPA min/week post-escalation weeks 9–12. Of the 9 women in cohort 2 continuing the intervention to 24 weeks, 7 lost ≥ 25% VAT (from baseline); these 7 had a median MVPA of 231 min/week at week 24.

### 3.7. Change in Systemic Biomarkers

After 12 weeks of intervention, significant improvement was observed for insulin, adiponectin:leptin ratio, omentin-1, leptin, SHBG, and CRP (all *p* < 0.05 by univariate analyses Table 5). At 12 weeks there were also numerical but non-significant decreases in FGF2, IL6, and MCP-1. Median adiponectin:leptin ratio (a marker of adipose function and insulin sensitivity) improved from 0.77 (below normal) to 1.08 (normal) [56]. In the 9 women who completed an additional 12 weeks in cohort 2 (24 weeks total) there was continued significant improvement in leptin and adiponectin:leptin ratio (*p* = 0.025), a significant decrease (−70%) in FGF2 (*p* = 0.018), and numerical improvement in insulin and CRP (data not shown). After 12 weeks of intervention, women losing >10% of their initial weight had significantly greater increases in the adiponectin:leptin ratio (*p* = 0.010), and decreases in leptin (*p* = 0.008), than did those losing <10%. 

### 3.8. Association of VAT Loss with Improvement in Adipokines and Fitness

**Figure 5:**
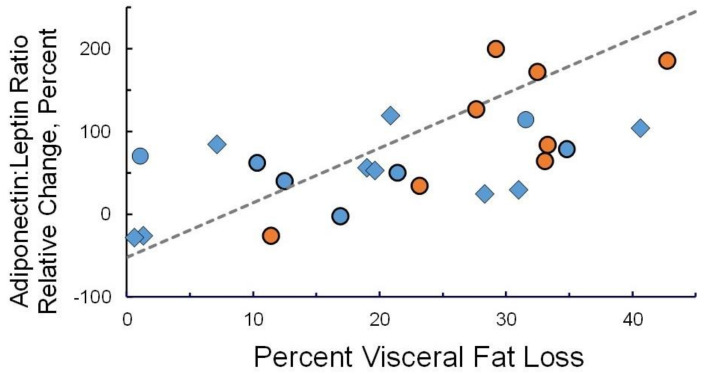
Change in serum adiponectin:leptin ratio at 12 and 24 weeks as a function of visceral fat lost, both relative to baseline values. Diamonds denote participants in cohort 1; circles denote participants in cohort 2 (blue = 12-week values; orange = 24-week values). Linear regression yields a slope of 6.6% change in adiponectin:leptin ratio per percent visceral fat loss (*p* < 0.001).

VAT loss after 12 weeks of intervention was associated with significant improvement in multiple markers including adiponectin:leptin ratio (*p* = 0.006), omentin-1 (*p* = 0.008), FGF-21 (*p* = 0.015), and resistin (*p* = 0.045). There was a linear correlation between VAT loss at 12 and 24 weeks and the adiponectin:leptin ratio at that time (Figure 5).

The strongest correlation (Spearman’s rho) for change in the adiponectin:leptin ratio was with VAT loss and not with other variables in Table 2 (i.e., total mass, lean mass, fat mass, fat mass index). 

## 4. Discussion

Our study demonstrated the feasibility of rapid increases in MVPA to a high level (≥ 200 min/week) over a short interval during caloric restriction in obese poorly fit postmenopausal breast cancer survivors. The 12 weeks of partially supervised exercise with twice-weekly personal trainers plus a home exercise prescription is similar in concept and duration to many third party-covered rehabilitation programs after a major cardiac event. The 2-cohort design was meant to preliminarily assess the likelihood of maintaining high MVPA levels after discontinuing the study-provided trainer but continuing the behavioral intervention and access to exercise facilities. At a relatively short follow-up of 12 weeks after trainer services discontinuation this also appeared favorable. 

Supervision has been shown to produce better results than autonomous exercise in obese breast cancer survivors; but long-term supervision is not feasible for the majority in the community setting [57]. Our achieved MVPA volume is similar in magnitude to that in other supervised exercise studies in breast cancer survivors without concomitant caloric restriction [28]. Our median VAT reduction of 20% with corresponding mass loss of 8% after 12 weeks of modest caloric restriction and partially supervised exercise is similar to reports in postmenopausal women without breast cancer [58]. Brown et al. recently reported a trial in overweight and obese breast cancer survivors randomized to either caloric restriction, supervised small group aerobic and resistance exercise, both, or control [59]. The supervised exercise groups were conducted weekly for 6 weeks then monthly for the remainder of the 12-month study. Weekly exercise goals were 180 min per week and MVPA was self-reported vs. measured as in the current study [60]. Reduction in weight (~7%) and VAT (~ 17%) at 12 months in their combined diet and exercise arm was similar to our results at 12 weeks; however, randomization to the combination arm did not improve either weight or VAT reduction over caloric restriction alone [59]. This result is different from prior reports, which may be a function of lower MVPA volume [32,33]. 

The improved systemic adipocytokine profile (increased adiponectin:leptin ratio, increased omentin, decreased insulin) observed after only 12 weeks has implications for breast cancer risk and recurrence [61,62,63,64]. Insulin and leptin are mitogenic, and leptin promotes angiogenesis and stem cell renewal [17,65]. Adiponectin antagonizes the pro-inflammatory and pro-oncogenic activity of leptin in addition to promoting insulin sensitivity [62]. Our observed normalization of the adiponectin:leptin ratio in the majority of participants is an indicator of improved metabolic function [56,66]. Omentin-1, which has anti-inflammatory, insulin sensitizing, and tumor suppressive properties, is produced primarily by VAT and its secretion is increased with exercise training [63,67,68]. FGFs are also thought to be secreted primarily by VAT and FGF-binding and activation of FGF receptors by has been associated with resistance to endocrine therapy [69,70]. FGF2 and FGF21 both appeared to be reduced by the weight loss intervention in our study although this was not significant for FGF2 at 12 weeks or FGF21 at 24 weeks. 

We did not observe significant reductions in the inflammatory cytokines IL-6 and TNFα, and only a borderline reduction in CRP. Although potentially implicated in breast cancer recurrence in obese individuals [71], reduction in inflammatory cytokines is not a consistent finding in behavioral weight loss trials [72]. Those incorporating long duration or high intensity exercise are most likely to report little reduction in pro-inflammatory cytokines [73,74]. Serum fatty acid binding protein-4 (FABP4) has been reported as higher in obese compared to lean breast cancer survivors [75] and is thought to play a role in breast cancer progression by facilitating provision of fatty acids to cancer cells [76]. We did not observe a decrease in FABP4 in our study; in fact some participants exhibited an increase in FABP4 despite weight and fat loss, possibly due to ongoing lipolysis with aerobic exercise [77]. Finally, a lack of decrease in bioavailable estrogen and testosterone, despite increases in sex hormone binding globulin (SHBG), may be due to use of aromatase inhibitors by the majority of our participants.

The widespread availability of YMCA facilities and YMCA contracted personal trainers makes our intervention highly translatable to the community setting. Rates vary from facility to facility but at the present time the full cost of a 3-month YMCA membership in the Kansas City area is ~USD 165 which generally includes group exercise and water fitness classes. Many Medicare plans and commercial insurance carriers will cover at least a portion of an exercise facility membership and behavioral counseling for weight loss [78]. The cost of the self-monitoring tools was USD 370 (Vivoactive 3^®^ smartwatch plus a chest strap at USD 270, and wireless digital scale USD 110). The 12-week duration of partially supervised exercise and low cost of USD 45 per supervised session makes trainer services affordable at USD 540.

A strength of our investigation is that activity trackers with heart rate monitoring were continuously worn by participants, allowing for objective measures of MVPA rather than self-reported measures that are less reliable. This allowed us to accurately correlate changes in MVPA with change in body composition and systemic risk biomarkers. Another strength was the use of 24 h recalls to assess dietary intake, which is the gold standard for dietary assessment. Limitations of our study include the small sample size, lack of objective measure of MVPA or caloric intake prior to enrolling on study, and difficulty in discerning the relative influence of caloric restriction vs. exercise on VAT and systemic biomarkers. While the gold standard for dietary assessment, 24 h recalls may be subject to recall bias; for example, NHANES data demonstrated that obese women underreported caloric intake by almost 900 kcal/day [79]. Further, women who are most compliant with caloric restriction may be most compliant with exercise. This was a feasibility study and correlations of the intervention with change in body composition and systemic biomarkers were exploratory and not corrected for multiple comparisons. Consequently, our findings need to be considered hypothesis generating rather than conclusive. 

Greater accuracy in estimation of caloric intake and energy expenditure can be achieved in free living individuals with double-labeled water and DXA body composition measures in conjunction with a recently developed formula [80]. Future studies incorporating these improved measures can facilitate development of algorithms for estimating caloric restriction, given volume and intensity of exercise and desired fat/visceral fat loss.

## 5. Conclusions

Despite our study limitations, we have demonstrated the ability to rapidly escalate MVPA over 9 weeks to ≥200 min/week in older sedentary obese breast cancer survivors undergoing a behavioral weight loss intervention using partially supervised exercise in convenient YMCA locations. Adherence was excellent in both cohorts. MVPA volume was correlated with substantial visceral fat loss and improved adiponectin:leptin ratio profile by 12 weeks. High volume MVPA appeared to be sustainable over an additional 12 weeks with discontinuation of study provided personal trainer but continuation of the behavioral intervention and provision of the exercise facility. The wide availability of YMCA facilities and associated personal trainers and short-term use of supervised exercise makes this intervention applicable to the community setting. Further testing in a larger cohort with more precise measures of caloric intake and energy expenditure plus a longer follow-up period is warranted.

## Figures and Tables

**Table 1:** Baseline characteristics of 22 participants in the intervention.

Variable	Median (Range) or *n* (%)		
	Survivors *n* = 18	High Risk *n* = 4	Total *n* = 22
Age, y	60 (40−70)	57 (55−60)	60 (40−70)
Race		4 (100%)	
Caucasian	16 (89%)		20 (91%)
African American	2 (11%)		2 (9%)
Time since diagnosis, months	27.5 (12−125)	NA	27.5 (12−125)
Prior chemotherapy	12 (67%)	NA	12 (55%)
Current anti-hormone use	12 (67%)	0 (0%)	12 (55%)
Current statin use	4 (22%)	0 (0%)	4 (18%)
Current aspirin use	7 (39%)	3 (75%)	10 (45%)
Current ACE inhibitor use	5 (28%)	0 (0%)	5 (23%)
Height, cm	164 (158−178)	159 (157−161)	163 (157−178)
Weight, kg	96 (77−126)	91 (79−107)	96.0 (76.5−125.7)
BMI, kg/m^2^	37.0 (30.8−43.3)	35.8 (31.7−41.1)	37.0 (30.8−43.3)
Waist circumference, cm	107 (94−135)	106 (91−110)	107 (91−135)
VO_2peak_, mL/kg/min	18.7 (13.7−25.3)	19.9 (18.0−21.0)	18.9 (13.7−25.3)
DXA Total Mass, kg	95.0 (75.9−125.3)	89.9 (77.5−104.9)	97.5 (75.9−125.3)
Lean Mass, kg	47.6 (39.2−57.0)	43.9 (36.6−46.7)	46.7 (36.6−57.0
Fat Mass, kg	47.6 (33.8−65.9)	44.5 (38.7−56.5)	47.6 (33.8−65.9)
Fat Mass Index, kg/m^2^	17.6 (13.2−23.0)	17.5 (15.6−21.8)	17.6 (13.2−23.0)
Visceral Adipose Tissue, kg	1.74 (0.98−2.95)	2.13 (1.42−2.92)	1.78 (0.98−2.95)

BMI = body mass index. No statistically significant differences between participant type by Mann–Whitney non-parametric test; except height (*p* = 0.019).

**Table 2:** Changes in physical activity, energy intake and morphometric (iDXA) parameters for 22 women completing the 12-week weight-loss intervention which included twice weekly personal trainer sessions. Median (range) values. *p*-values from Wilcoxon signed rank test, 2-tailed.

Variable or Category	Baseline	12 Weeks	Change	Relative Change, %	*p*-Value for Change
VO_2peak_, mL/kg/min	18.9 (13.7–25.3)	22.0 (17.1–31.4)	2.7 (−1.0–9.6)	13 (−5–44)	<0.0001
Energy intake (2-day food recall), Kcal (cohort 2 only)	1522 (1092–3383)	1321 (851–1913)	−348 (−2369–308)	−25 (−70–28)	0.041
MVPA min/week (Zone 3–5 recorded Garmin Connect); median for weeks 9–12	55 (week 1) (0–226)	219 (56–353)			0.0001
BMI, kg/m^2^	37.0 (30.8–43.3)	32.5 (29.5–41.3)	−2.7 (−7.2–0.2)	−7 (−20–1)	<0.0001
Waist Circumference, cm	106.5 (91–135)	99.5 (89–124)	−6 (−22–6)	−5 (−18–0)	<0.0001
DXA Total Mass, kg	95.0 (75.9–125.3)	94.0 (73.0–118.9)	−7.7 (−20.5–0.4)	−8 (−20–0)	<0.0001
Lean Mass, kg	46.5 (36.6–57.0)	44.8 (37.3–56.5)	−1.6 (−4.7–1.6)	−3 (−9–3)	0.0019
Fat Mass, kg	47.6 (33.8–65.9)	39.2 (32.1–59.3)	−6.2 (−15.6–−1.2)	−13 (−33–−2)	<0.0001
Fat Mass Index, kg/m^2^	17.6 (13.2–23.0)	14.9 (11.8–20.5)	−2.3 (−5.7–−0.4)	−13 (−33–−2)	<0.0001
Visceral Fat Tissue, kg	1.74 (0.98–2.95)	1.36 (0.77–2.50)	−0.37 (−0.95–−0.01)	−19 (−41–−1)	<0.0001

**Table 3:** Changes in V0_2peak_, energy intake, anthropomorphic, and body composition (DXA) parameters, restricted to 18 breast cancer survivors completing the 12-week weight-loss intervention which included twice weekly personal trainer sessions. Median (range) values. *p*-values from Wilcoxon signed rank test, 2- tailed.

Variable or Category	Baseline	12 Weeks	Change	Relative Change, %	*p*-Value for Change
VO_2peak_, mL/kg/min	18.7 (13.7–25.3)	21.1 (17.1–31.4)	2.3 (−1.0–9.6)	12 (−5–44)	0.0003
Energy intake (2-day food recall), Kcal (cohort 2 only)	1522 (1092–3383)	1381 (851–1913)	−233 (−2369–308)	−14 (−70–28)	0.24
BMI, kg/m^2^	37.0 (30.8–43.3)	32.5 (29.5–41.3)	−2.4 (−7.2–0.2)	−7 (−20–1)	0.0002
Waist Circumference, cm	106.5 (94–135)	99.5 (89–124)	−6 (−22–−1)	−5 (−18–−1)	0.0002
DXA Total Mass, kg	95.0 (75.9–125.3)	84.0 (74.1–118.9)	−7.4 (−20.5–0.4)	−7 (−20–0)	0.0002
Lean Mass, kg	47.6 (39.2–57.0)	45.3 (37.3–56.5)	−1.8 (−4.7–1.6)	−3 (−9–3)	0.0050
Fat Mass, kg	47.6 (33.8–65.9)	39.2 (32.1–59.3)	−6.2 (−15.6–−1.2)	−13 (−33–−2)	0.0002
Fat Mass Index, kg/m^2^	17.6 (13.2–23.0)	14.9 (11.8–20.5)	−2.2 (−5.7–−0.4)	−13 (−33–−2)	0.0002
Visceral Fat Tissue, kg	1.74 (0.98–2.95)	1.36 (0.77–2.34)	−0.37 (−0.95–0.01)	−20 (−41–−1)	0.0002

**Table 4:** Changes in VO_2peak_, anthropomorphic, and body composition (DXA) parameters for 9 women complementing a 24-week weight-loss intervention with twice weekly personal trainers during initial 12 weeks only but provided continued access to exercise facility weeks 13–24. Median (range) values. p-values from Wilcoxon signed rank test, 2-tailed.

Variable or Category	Baseline	12 Weeks	Relative Change, % 0–12 Weeks †	24 Weeks	Change 0–24 Weeks	Relative Change, % 0–24 Weeks	*p*-value for Change 0–24 Weeks
VO_2peak_, mL/kg/min	18.0 (13.7–25.3)	21.7 (17.4–28.9)	16 (7–28)	22.9 (20.2–31.0)	4.8 (3.5–10.0)	26 (17–48)	0.012
BMI, kg/m^2^	33.6 (31.7–42.8)	31.7 (29.5–36.8)	−9 (−17–−3)	30.9 (28.2–37.0)	−4.7 (−7.1–−1.0)	−12 (−17–−3)	0.008
Waist Circumference, cm	104 (91–120)	103 (90–114)	−5 (−7–0)	102 (89–109)	−7 (−15–−2)	−6 (−14–−2)	0.007
DXA Total Mass, kg	91.9 (77.5–116.1)	81.8 (73.0–102.3)	−8 (−22–−2)	81.5 (68.9–97.7)	−11.8 (−21.6–−2.2)	−12 (−19–−3)	0.008
Lean Mass, kg	43.1 (36.6–54.3)	43.0 (37.6–55.5)	−3 (−5–3)	43.6 (36.9–52.9)	−1.4 (−2.8–0.9)	−3 (−6–2)	0.066
Fat Mass, kg	49.3 (38.7–62.5)	40.1 (32.5–53.9)	−14 (−19–−8)	39.5 (29.3–47.8)	−9.6 (−14.8–−2.5)	−20 (−24–−6)	0.008
Fat Mass Index, kg/m^2^	17.6 (15.5–23.0)	14.6 (13.1–19.8)	−14 (−19–−8)	14.8 (11.8–17.5)	−3.7 (−5.4–−1.0)	−20 (−24–−6)	0.008
Visceral Fat Tissue, kg	1.64 (0.98–2.92)	1.32 (0.77–2.50)	−19 (−35–−1)	1.26 (0.73–2.07)	−0.54 (−0.85–−0.16)	−29 (−43–−11)	0.008

† All comparisons of 12 week to baseline values for the subset of 9 participants are statistically significant (*p*-value range 0.003 to 0.041).

**Table 5:** Serum biomarker changes for 22 women completing the 12-week weight loss intervention. Median (range) values. *p*-values from Wilcoxon signed rank test, 2-tailed.

Biomarker	Baseline	12 Weeks	Rel Change, %	*p*-Value
Adiponectin, μg/mL	26 (6–218)	28 (7–216)	8 (−42–89)	0.59
Leptin, ng/mL	38 (17–60)	24 (5–48)	−30 (−81–146)	0.001
Adiponectin:Leptin Ratio, μg/mL:ng/mL	0.8 (0.1–10.7)	1.1 (0.2–15.7)	55 (−28–699)	0.001
Lipocalin-2, ng/mL (cohort 2 only)	116 (42–132)	116 (47–123)	−1 (−42–111)	0.93
Resistin, ng/mL	22 (11–32)	24 (12–37)	6 (−57–96)	0.76
PAI-1, ng/mL	78 (13–122)	80 (12–101)	−10 (−40–124)	0.31
IL-6, pg/mL	3.0 (0.9–12.2)	2.3 (0.9–22.0)	−7 (−61–194	0.36
Insulin, pg/mL	341 (100–2196)	314 (47–1480)	−17 (−60–266)	0.012
HGF, pg/mL	206 (42–641)	221 (61–363)	−4 (−58–132)	0.28
MCP-1, pg/mL	322 (97–493)	299 (142–675)	−3 (−30–158)	0.86
TNFα, pg/mL	4.2 (1.9–11.2)	4.6 (1.8–11.8)	5 (−39–70)	0.31
CRP, μg/mL	8.1 (1.2–87.4)	6.3 (0.7–255.2)	−25 (−75–167)	0.048
FGF-2, pg/mL	2.0 (0.3–15.0)	1.2 (0.3–4.0)	−28 (−89–323)	0.23
FGF-21, pg/mL	287 (45–894)	259 (34–640)	−6 (−57–146)	0.46
Lysyl oxidase, ng/mL (cohort 1 only)	2.0 (0.8–3.7)	2.3 (0.0–4.9)	−11 (−100–214)	0.42
Visfatin, ng/mL	51 (24–68)	54 (27–64)	2 (−14–40)	0.32
Omentin, ng/mL	2.8 (1.8–23.9)	3.0 (1.9–21.3)	7 (−10–113)	0.033
FABP4, ng/mL	54 (20–89)	59 (16–131)	19 (−26–93)	0.062
Estradiol, pg/mL	77 (33–138)	69 (36–174)	4 (−30–47)	0.44
Estradiol, pmol/L	0.3 (0.1–0.5)	0.3 (0.1–0.7)	4 (−30–47)	0.46
Testosterone, ng/mL	1.2 (0.3–4.9)	1.1 (0.3–2.8)	7 (−82–48)	0.26
Testosterone, nmol/L	4.1 (1.1–16.9)	3.9 (1.2–9.7)	7 (−82–48)	0.26
SHBG, nmol/L	36 (20–231)	40 (22–208)	10 (−12–80)	0.044
Free Estradiol, pmol/L	4.4 (0.6–9.7)	3.9 (1.0–9.1)	−5 (−42–67)	0.83
Free Testosterone, pmol/L	79 (11–327)	62 (17–166)	−5 (−83–53)	0.14

## Data Availability

Datasets with individual subject (deidentified) results may be supplied upon reasonable request to the corresponding author.
